# Exploring the experiences of clients receiving opioid use disorder treatment at a methadone clinic in Kenya: a qualitative study

**DOI:** 10.1186/s13722-022-00352-z

**Published:** 2022-12-12

**Authors:** Sarah Kanana Kiburi, Jackline Mwangi, Geoffrey Maina

**Affiliations:** 1 Department of Medicine, Aga Khan University Hospital, Nairobi, Kenya; 2 Discipline of Psychiatry, University of KwaZulu Natal, Durban, South Africa; 3 Department of Psychology, Kenyatta University, Nairobi, Kenya; 4 College of Nursing, Prince Albert Campus, University of Saskatchewan, Prince Albert, Canada

**Keywords:** Client lived experiences, Opioid use disorder, Methadone treatment, Kenya

## Abstract

**Background:**

Assessing the experiences of individuals on methadone treatment is essential to help evaluate the treatment program’s effectiveness. This study aimed to explore the experiences of patients receiving methadone treatment at a clinic in Nairobi, Kenya.

**Method:**

This study employed an exploratory qualitative study design. Through purposive sampling, participants were enrolled from individuals attending a methadone clinic for at least 2 years. Semi-structured individual interviews were used to collect data on substance use and experience before methadone treatment and experiences after starting methadone treatment, including benefits and challenges. Interviews were transcribed, and NVIVO 12 software was used to code the data using the preidentified analytical framework. Thematic analyses were utilized to identify cross-cutting themes between these two data sets. Seventeen participants were enrolled.

**Results:**

Seventeen participants were enrolled comprising 70% males, with age range from 23 to 49 years and more than half had secondary education. The interview data analysis identified four themes, namely: (a) the impact of opioid use before starting treatment which included adverse effects on health, legal problems and family dysfunction; (b) learning about methadone treatment whereby the majority were referred from community linkage programs, family and friends; (c) experiences with care at the methadone treatment clinic which included benefits such as improved health, family reintegration and stigma reduction; and (d) barriers to optimal methadone treatment such as financial constraints.

**Conclusion:**

The findings of this study show that clients started methadone treatment due to the devastating impact of opioid use disorder on their lives. Methadone treatment allowed them to regain their lives from the adverse effects of opioid use disorder. Additionally, challenges such as financial constraints while accessing treatment were reported. These findings can help inform policies to improve the impact of methadone treatment.

**Supplementary Information:**

The online version contains supplementary material available at 10.1186/s13722-022-00352-z.

## Introduction

Opioid use is prevalent worldwide, with a global twofold increase between 2010 and 2020 and is associated with a significant burden of disease and mortality globally [1]. For example, more than 75% of deaths from substance use disorders are attributed to opioid use and an estimated 109 500 people died from opioid overdose in 2017 globally [2, 3]. Opioid use disorder (OUD) also causes adverse health effects with significant economic burdens due to the cost of care and lost productivity [2, 4–6]. While there is a shortage of research on opioid use prevalence in Africa, it is an emerging problem with a significant impact on opioid use [7]. In Kenya, the current prevalence of opioid use ranges from 0.1% in the general population in 2017 [8] to 4.5% among inpatients on treatment for substance use disorders [9].

Treatment for OUD includes psychosocial treatment and pharmacotherapy [10]. Medications for opioid use disorder (MOUD) include methadone, buprenorphine and naltrexone, of which methadone is the commonest. A systematic review of MOUD provision globally showed that methadone was most commonly prescribed in 81out of 86 countries, while buprenorphine was used in 56 countries, out of which 52 also prescribed methadone [11]. However, despite the reported benefits, use of MOUD globally is suboptimal [3, 11, 12]. Research among patients on methadone treatment has reported a wide range of experiences. Benefits reported include improved quality of life, sleep, and appetite and a positive impact of treatment [13]. Conversely, financial barriers, lack of awareness, negative attitudes towards methadone treatment, social stigma and poor access to treatment facilities are barriers that impact MOUD uptake [14].

In Africa, MOUD is available in ten countries, and there is limited research on clients’ experiences with methadone treatment [15]. The use of low-threshold methadone treatment in South Africa increased retention in the care [16] and a study on the provision of comprehensive services in Tanzania optimized the effectiveness of the methadone treatment [17]. In Kenya, methadone treatment gave clients hope and aspiration in their recovery journeys [18].

In Kenya, it is estimated that there are 18,000 to 30,000 injecting drug users, most of whom use heroin with an HIV prevalence more than three times that of the general population [19]. This prompted the government in association with partners to introduce methadone treatment as a harm reduction strategy in 2014 in Nairobi [20, 21] with current eight methadone treatment clinics in various regions of the country. In addition to methadone treatment, the clinics offer comprehensive care that includes treatment for other substance use and other co-occurring disorders.

Limited research has been done to assess the implementation of methadone treatment in Kenya. Previous studies among individuals on methadone treatment include a study at a community-based drop-in centre related to client perception of accessibility to methadone treatment [18]; understanding of the integration of HIV services with methadone [21]; assessment of transition from smoking to injection drug use [22]; impact of needle syringe exchange program on injection drug use [23]; and effect of methadone treatment on ART uptake and viral suppression among people who inject drugs [19]. However, no study in Kenya has described the experiences of patients receiving methadone treatment at a methadone treatment clinic. Assessing the experiences of individuals on methadone treatment is essential to help evaluate the treatment program to improve the quality of services and treatment outcomes and reveal the perception of MOUD and the clinic environment as these factors may influence adherence and retention in the treatment [24, 25]. The aim of this study was to explore the experiences of patients receiving methadone treatment at a Nairobi clinic.

## Methods

### Study setting and participants

This study employed an exploratory qualitative study design to explore clients’ experiences of seeking treatment for opioid use disorder. This study was based at a Methadone treatment clinic in Nairobi County. The clinic is one of the country’s eight government-owned methadone treatment clinics offering treatment individuals with OUD. Those enrolled in the study were: aged 18 years and older, medically stable, able to consent, and enrolled at the methadone treatment clinic for at least 2 years.

### Sampling and recruitment procedure

Purposeful sampling was employed to recruit participants while ensuring sociodemographic diversity of participants and allow recruitment across ages and gender. Written consent was obtained before the interviews were conducted. Two researchers, SK and JM, conducted the interviews using semi-structured interview guides. Interviews were conducted once for each participant between March 2020 and August 2020, which coincided with national public health measures to contain COVID-19 infection. Because the clinic restricted access to clients, only essential services were offered. Research activities were therefore suspended until public health measures for the COVID-19 pandemic were relaxed to allow for the face to face interactions.

These interviews, either in English or Kiswahili, were recorded with a digital voice recorder for transcription. The questions in the interview guide included: sociodemographic characteristics such as age, gender, education and marital status; substance use before starting treatment; experiences at the methadone clinic, including perceived benefits, access to care and challenges faced; social support; and factors associated with having dropped out of treatment. A summary of questions contained in the interview guide is attached as Additional file 1: Table S1 (Appendix 1). The interviews lasted 10–20 min. This was due to limitation in time for the participants who were in a hurry to get back to work after their daily visit to the clinic.

### Data analysis

Interviews conducted in Kiswahili were translated during the transcription stage by a research assistant, an undergraduate psychology student not affiliated with the clinic. SK (lead researcher) verified the accuracy of the translation by listening to the audio recording and reviewing the interview transcripts. An analytical framework was developed using interviews with the participants based on the process identified by Gale and colleagues [26]. These steps include: Familiarization with the data whereby a couple of interviews were read several times to identify preliminary threads and patterns that emerged from the data. Next open coding was performed on three rich transcripts, after which the nodes were aggregated and refined to form an analytical framework. This framework was used to analyze the remainder of the interviews using NVIVO 12 software. Thematic analysis was then applied to identify cross-cutting themes by consolidating the created nodes, which are relevant passages in a coded transcript. They can represent a code, a theme, or an idea about the data in a project [27]. An inductive approach was used to guide the identification of nodes clustered into themes.

## Results

Table 1 summarizes the sociodemographic variables of the 17 participants enrolled in this study. The majority were males (70.6%) and aged between 21 and 30 years (41.2%) with an age range of 23 to 50 years, and the duration of treatment ranged from 11 to 36 months. Participants started using opioids during their teenage years to early adulthood and were preceded by other drugs, especially marijuana and cigarettes, that participants were exposed to early. Most participants were introduced to substances by people close to them, such as family members, friends, peers, or close relatives.

**Table 1:** Sociodemographic characteristics of study participants

Variable	Frequency	Percentage (%)
Age		
21–30	7	41.2
31–40	4	23.5
> 40	6	35.3
Sex		
Male	12	70.6
Female	5	29.4
Marital status		
Single	5	29.4
Married	8	47.1
Separated	4	23.5
Education		
None	1	5.9
Primary	9	52.9
Secondary	7	41.2
Age at first substance use (any substance) in years		
Less than 10	1	5.9
10–15	5	29.4
16–20	9	52.9
More than 20	2	11.8
Substance first used		
Cannabis	10	58.8%
Cigarette	3	17.6%
Benzodiazepines	1	5.9%
Khat	2	11.8%
Heroin	1	5.9%
Age at first opioid use		
10–15	2	11.8%
16–20	5	29.4%
More than 20	10	58.8%
Route of opioid use		
Smoking	11	64.7%
Injecting	6	35.3%
Duration in methadone treatment in months		
Less than 12	3	17.6%
13–24	5	29.4%
24–36	9	53.0%

### Themes identified

Four themes were identified from data analysis: (a) the impact of opioid use before starting methadone treatment; (b) learning about methadone treatment; (c) experiences with care at the methadone treatment clinic; (d) barriers to optimal methadone treatment. A summary of the themes is shown in Table 2.

**Table 2:** Summary of themes identified in the study

Main theme	Category
Impact of opioid use before treatment	Physical Health (Loss of appetite, poor feeding, discomfort from withdrawal symptoms, increased HIV risk)
	Self-care neglect (dirty, unkempt)
	Financial losses (Job and business loss, spending huge amounts of money on heroin)
	Family dysfunction (Separation from spouse, running away from home)
	Interaction with law enforcements (Due to stealing and shop lifting)
Learning about methadone treatment	Civil society organizations (outreach activities, use of incentives)
	Family and friends who had improved after starting methadone treatment
Experiences at the methadone clinic	Physical health (Improved health, appetite and feeding, improved self-care)
	Restoration of broken relationships (Reunion with spouse and other family)
	Improved finances (Better saving; no money used to buy drugs; use money for self-improvement)
	Improved social relations (Less stigmatization, Contributing to society)
Barriers to optimal methadone treatment	Financial (Requires bus fare to travel to clinic)
	Long distance (From home to the clinic takes time)
	Strict rules (Frequent discontinuation if a client break treatment contract)
	Fixed opening hours (Inaccessibility when needed by clients especially those who work)
	Lack of holistic care (Focus only on treatment and doesn’t address socioeconomic needs)

### Theme 1: impact of opioid use before starting methadone treatment

Participants reported that opioid use significantly impacted their physical, social, and economic aspects. As a result of opioid use, most participants reported neglecting self-care and activities of daily living, such as showering and doing laundry. Subsequently, their friends avoided them because of their unkempt appearance and foul body odor.

*“They used to hate me because I looked dirty, and I used to stink; therefore. They knew that I was using something bad and that I was on drugs” *(Male, 47).

*“That thing has destroyed my life in many ways; it made me separate from my wife, sleep outside, and became hopeless. It destroyed my life because when I separated from my wife, I used to sleep outside and became very dirty”* (Male, 50)

Due to opioid use, participants health suffered significantly from lack of appetite, poor eating habits, and unhealthy eating. Others spent their income on drugs at the expense of food, and as such, they suffered hunger or survived on unhealthy food such as cakes, biscuits, and juice leading to poor health over time.

*“Eating was a problem that is why you see the drug users are very skinny. The appetite seems to vanish whenever you use the drug and comes back when the money is finished” *(Male, 47)*.*

Gastrointestinal symptoms such as diarrhea and nausea related to withdrawal from opioids were also responsible for the worsening health of most participants who could not always afford to use opioids every day. Until recently, since there was no treatment for opioid use disorder, clients presenting at health facilities were either misdiagnosed or treated for other illnesses such as malaria, whose symptoms were often confused with withdrawal symptoms.

*“There was a time I had gone upcountry due to how stuff had affected me. To avoid it while I was there, I only stayed a day and fell ill. I was taken to hospital and diagnosed with malaria, but I told them what I was using, and they could not help me beyond that, so I went back to the den” *(Male, 23)*.*

When left untreated, opioid use disorder increases the user’s risk for blood-borne infections such as HIV/AIDS from sharing used needles and engaging in unprotected sex. Poorly disposed needles increase the risk of contracting a blood-borne infection due to environmental hazards.

*“Heroin use made me find myself in situations I never expected. Although I never injected heroin, I used it with people who did it. It was not uncommon to find used syringes carelessly disposed of on the ground, increasing the risk of needle pricks. Other times, some users used dirty needles as a weapon to threaten or prick someone when in a fight. I am certain my HIV infection is not due to sex with men but unsafe needle disposal”* (Female, 25).

Women were more vulnerable to sexual exploitation in exchange for opioids. Others kept multiple sexual partners to sustain their expensive heroin addiction, and they risked unwanted pregnancies and infection with HIV*.* Because of a lack of strong social support, such women started families on the street.

*“Girls who are into heroin are exploited and violated by the men to finance their heroin use, an expensive drug. Desperate for a fix, a man would offer to share his supply with you in exchange for sexual favours” *(Female, 25)*.*

Opioid use also predisposed users to engage in unlawful behaviours such as stealing to fund their OUD leading to frequent arrests. Moreover, opioid use attracted significant stigma from society, and users were often treated with suspicion, regularly interrogated, and denied access to shops and malls. Others experienced hostility in public and have been deemed a menace to society. Subsequently, many respondents lived isolated to limit contact with the public, while others ran away from home to be homeless.

*“Because of stealing, you are always at loggerheads with other people you steal from. That is why you find people with their own homes leaving and opting to do all their stuff from the streets. They eat in the streets, steal, and go back to the streets, and sleep there. You live like a homeless person, yet you have a place to live. I had also left home but later went back” *(Male, 21)*.*

Opioid use significantly impacted family and social relations. Participants expressed a common concern of constant quarrelling with parents, siblings, and significant others due to opioid use. Family conflict also arose when participants sold family property such as cell phones and household goods to fund their opioid use disorder. Besides family infights, some participants were estranged from their families through marriage dissolution or separation due to substance use.

*“Like my parent (father) when he heard that I had come here and indulged myself in bad things like smoking heroin, he alienated me. Like they didn’t even tell me when my mum passed on, but when I started methadone treatment, I even got their numbers, and we started communicating that's when they even told me that my mum had passed* on” (Female, 25).

Those that had been exposed at an early age to opioids were unable to continue with their education. Therefore, many did not possess formal academic credentials, which hindered their ability to find decent jobs. Those with a decent source of income wasted them on substances and were thus unable to invest, save, or hold on to jobs. Such clients were dismissed from work for engaging in illegal activities like stealing from a client and employer or due to performance issues while others sold personal items at a throwaway price to afford opioids.

*“When I started taking heroin, I used to take my business items and sell them. Sometimes I would sell them at low prices compared to how much I had purchased them. The people who used to give me work realized that I would steal some parts when they bring their cars, and I started losing jobs.” *(Male, 34).

OUD had significant social, economic, and health impacts that hindered participants’ ability to participate fully in society. As ostracized citizens, they lived on the margins of society with minimal hope.

### Theme 2: learning about methadone treatment

Participants learned about the methadone treatment program, from friends and family members and community outreach events. Outreach programs were the commonest channels through which these participants were recruited to the methadone treatment program. These drop-in centers are run by civil society organizations that reach out to persons with substance use disorder, conduct the initial screening and other harm reduction strategies, and refer those willing to start methadone treatment. Incentives, such as free food and training, were provided to attract heroin users to the methadone program.

*“There were some doctors who used to come in the streets and give us milk and bread and tell us that a drug has been brought that would help us quit taking heroin. They also said it would be nice if we reformed and came for classes where we would be taught” *(Male, 41).

*“You go there and listen to what you are being taught…After observing how you are improving, they now set up a date and tell you when you will start taking methadone”* (Female, 25)

Some were introduced to the methadone treatment program by individuals who had stopped opioid use, who impressed them with the transformation that methadone brought to their lives. Such participants were amazed that their friends did not appear interested in using opioids anymore, heightening their curiosity about what change had happened in their lives and gave them motivation to seek treatment.

*“When we went back to town with my friend, I bought heroin and started smoking. Unlike in the past, he didn’t ask me for it; he was my partner and a close friend. The fact that I escort him to come here shows he is a dear friend. I wondered why he would not smoke heroin”* (Male, 41).

Others were encouraged to enroll in treatment by their relatives and family members who had prior experience with the methadone treatment program and its transformative impact.

*“My husband used to get methadone at (the other clinic). He always used to tell me to join methadone treatment because it was good, and he always insisted, but I did not think it would help because people used to say that methadone was just another drug.”* (Female, 23).

Although participants could see evidence of methadone use transformation among former opioid users, some were reluctant to join the methadone bandwagon. This was partly due to misinformation, bad advice, methadone stigma, and lack of knowledge about how methadone worked to treat heroin addiction.

*“I saw a friend who was on methadone treatment improving. She talked to me about the importance of methadone. We were told that methadone use would lead to sterility and even death but on research, I found out that they were all lies. I, therefore, went to (a drop-in Centre) and talked to the doctors there who directed me on how to start.”* (Female, 32).

Despite the discouragements and misinformation regarding methadone and its side effects, participants somehow pressed on to investigate it and eventually enrolled in it. They hence got a first-hand experience with the treatment.

### Theme 3: experiences with care at methadone treatment clinic

Most participants reported that methadone treatment transformed their lives. This transformation was evidenced by their improved appearance, physical health, and restoration of broken or lost relationships. With their lives changing, they experienced less stigma and discrimination from society. Some participants experienced this transformation within a short time of starting methadone treatment.

*“After 1 month of taking methadone, I had seen big changes. I used to sleep well. I have an appetite, sometimes I took a shower two to 3 days per week but now you see, the first day I took methadone, the next day I woke up in the morning at 5, I showered, went to the mosque and prayed, it's something I mean something I have never done”* (Male, 43).

Because methadone helped clients deal with withdrawal symptoms and cravings from opioid use, participants found it easy to stop using opioids. The monies they otherwise used to fuel their opioid use were redirected to a better cause, such as improving self-care and saving. Being able to earn an honest living was gratifying too.

*“We can see a lot of difference, and we have changed; we are clean, the money we used to smoke we don’t waste anymore. At least if you get even one hundred, you can buy some clothes and be smart like other people.”* (Female, 25).

The family restoration was an invaluable benefit participant got after their lives were stabilized by methadone treatment. Families were willing to take them in once they witnessed the difference that methadone treatment made in their lives.

*“I brought back my family because they had fled. I had been hated even by my mother and many people with me before I started taking heroin. They started ignoring and avoiding me. But now I tried and made other friends, I also avoided those we used to take drugs together, now am a different person”* (Male, 41).

The social stigma participants faced when they used opioids dissipated when they enrolled for methadone treatment and friends who had previously avoided them reconnected with them.

*“A lot has improved; people do not believe that I changed, and those who used to see me using heroin are ashamed of talking to me. Those who are no longer ashamed to talk to me do it with happiness. They usually ask me what changed, and I tell them it is methadone, something you are given, and you no longer feel like taking drugs” *(Male, 50)*.*

Methadone treatment stabilized their lives in a way that allowed them to restore their social connection, earn an honest living, and improve their health. Subsequently, they restored their lives and engaged in positive living as responsible citizens.

### Theme 4: barriers to optimal methadone treatment

Participants came from far distances to access the methadone treatment program. Most of the participants are unemployed and rely on casual jobs for their upkeep, so spending time working to seek treatment for OUD denied them opportunities to earn. Also, paying for bus fare to come to the clinic for daily methadone dosing under observation caused a financial strain which made some to miss clinic appointments or to skip a methadone dose. Some clients resulted in risky behaviours such as boarding cargo trucks to take them to the clinic, risking injuries from jumping off a fast-moving truck and others trekking long distances to the clinic, an exhausting and time-consuming exercise. Participants struggled to find a babysitter to leave their dependents with; therefore, coming with a baby to the clinic added to the logistical difficulties.

*“People are too many, and these services are very far, so you find people coming from very interior places to town. Some do not have the transport money, and you find that they hang on trucks to come here, risking their lives. Then you find that there are people who come from far and maybe on reaching the stage, it is almost time, so they resort to spending more on a motorcycle.”* (Male, 21).

Participants felt that enrolling in the methadone treatment program did not address other social challenges faced by most, such as homelessness. Street families constantly contended with hunger, poor hygiene, and environmental exposure. Such a lifestyle sabotaged their recovery process.

*“Methadone alone is not enough, and one is supposed to change their thoughts and behaviour as well as their ways of life, everything! …If a person stays in a place where they are stable, somewhere they do not have stress, they can reform very quickly. Somewhere with food, housing, they will reform very fast even if it is not giving them everythin.”* (Male, 21).

Many factors led participants to miss their methadone dose. These include apprehension by police for various misdemeanors, lack of bus fare, and inflexible work schedules. Missing any methadone dose eroded any recovery gain they had made in the treatment.

*“ If there is a way you can assist if someone misses, let’s say today if you go to the doctor tomorrow, if they can give you methadone rather than deny you, it would be helpful because when you leave there, you will hurt, and your mind will lead you to nowhere else than to heroin. So instead of telling me to go back to the streets, you better send me to a counsellor”* (Female, 25).

Because the clinic served a large catchment area, many clients experienced a prolonged waiting time due to overcrowding. Moreover, since the clinic was not operating outside of regular working hours, clients with limited working hours missed out on their doses. The challenges the clients experience in accessing care are related to the socioeconomic status of most clinic attendees and the nature of the clinic operation. Addressing these barriers can create a client-friendly environment that can increase clients' retention to care.

*“You can see, for example, when a person is late by just one minute or thirty seconds, they are just locked outside…. You are denied your dosage, and there is nowhere you can buy it; there is nothing you can do, yet you have decided to change. Like there is a guy who used to come here, I don’t know what he had done, but that is how he was expelled from the program, you see he had no option but to go back to drugs; if someone has decided to reform, they should not be denied this drug over petty issues that can be solved here”* (Male 21).

Personal and systemic factors outside the clients’ control presented significant barriers to treatment adherence, ultimately impacting the clients’ recovery journey.

## Discussion

This study aimed to assess the experiences of individuals attending a methadone maintenance treatment clinic in Kenya. Four themes were generated from the interviews focused on the impact of opioid use before starting methadone treatment to barriers to methadone treatment. In this study, it was noted that many participants started using opioids at an early age, causing them to drop out of school without attaining secondary education, without which, individuals were relegated to performing physically exhausting jobs or living below poverty levels. Education achievement is significant since it is associated with self-efficacy which influences onset and recovery from substance use ([28, 29]. Using substances early in life also strains relationships with relatives, which can hasten disconnection and social contact.

Given that the methadone program was introduced in Kenya in 2014, most individuals may have been exposed to opioids for an extended period which may have caused damage to their physical health. Therefore, when they seek to care for opioid use disorders, they not only suffer from the consequences of chronic heroin use but don't have the social capital required to support treatment retention and recovery. This further suggests the need for implementing substance use primary prevention strategies especially among the youth [30, 31].

Delay in seeking treatment for heroin addiction may have been attributed to the inability of the health care providers to correctly diagnose the addiction. There were reports that opioid withdrawal symptoms were often misdiagnosed as malaria, a common tropical disease in the country. Misdiagnosis of opioid use disorder and stigma toward clients with opioid use disorder contributes to a delayed referral of methadone treatment [32, 33]. This shows need for training non-specialists on screening and brief interventions for SUD in primary care settings [34, 35].

Community outreach centers were the most common referral for methadone treatment. To be enrolled in the MOUD program, individuals with OUD were required to be engaged in programs that offered harm reduction services such as needle exchange programs and other adjunct services [36]. This is similar to what has been reported previously [37]. Also, family and friends were reported to be conduits through which clients were enrolled in the methadone treatment. Lived experiences on the positive difference that methadone treatment made to clients with opioid use persuaded clients to consider MOUD as an option.

The lack of information about how methadone works and the stigma towards it was a significant barrier that prevented clients with OUD from enrolling in treatment. Moreover, stigma from self, family, friends and healthcare providers can hinder a client from seeking treatment, causing continued substance use [38]. Financial constraint was a significant barrier that hindered clients from attending the methadone treatment clinic as required, similar to what has been reported [14, 33]. Since most clients engage in casual jobs with unpredictable working hours, they do not always have the finances to afford a bus fare.

Homelessness was another significant impediment to continued retention in methadone treatment. Clients who lived on the street prioritized securing the necessities of life at the expense of seeking care for OUD. Therefore, they could not meet the demands associated with access to methadone treatment, including financial resources and time. Previous studies report that individuals living in unstable housing have adverse outcomes and reduced engagement with care compared to those with stable homes [39–41]. A long waiting time to enroll for methadone treatment was a significant barrier to care since the area has only two methadone treatment clinics. This shows a need to implement strategies that improve access to care.

Although methadone treatment is associated with reduced unlawful activities and interaction with the criminal justice system, those who persisted in these activities had their treatment interrupted due to incarceration. Provision of MOUD in criminal justice system is associated with better outcomes [42]. This is currently being implemented whereby methadone is being delivered to prison for clients incarcerated while receiving treatment at the clinic.

Despite the logistical challenges the clients experienced, they reported positive changes following methadone treatment, including reduced substance use, improved health, reconnection with family, less criminal activity, and increased productivity. This agrees with previous research on role of methadone treatment in recovery [43–46].

### Implication for practice

Findings from this study show benefits of methadone treatment as well as barriers that may lead to suboptimal clinical outcomes. To improve overall client well-being, certain practical measures can be undertaken. First, more healthcare providers need to be trained to diagnose OUD. Early diagnosis can foster linkage to care and provider a better treatment outcome for clients with complex needs associated with OUD [34, 35]. Also, once the clients are in the program, they need to be provided with comprehensive services that complement methadone treatment such as group therapy, motivational interviewing, and case management for individuals with opioid use disorder [47]. Additionally, peer interventions that are low-cost and effective in increasing retention and program satisfaction can be utilized in supporting clients on methadone treatment [48].

Secondly, OUD needs to be seen as a family problem as it affects the entire family of the one with OUD. Therefore, the family needs to be involved in their care. Inviting family members to accompany clients to the methadone treatment clinic can also dispel stigma [38]. Strong social support from family and friends has been shown to enhance treatment and retention to care as they constitute the social capital needed to support recovery [49, 50]. Public awareness campaigns on how methadone works as a treatment for opioid use disorder are required to counter stigma [38].

Fourthly, developing innovative solutions to dispense methadone treatment is required due to the unique socioeconomic challenges that hinder their access to treatment. This can be done by improving geographical access such as providing treatment in multiple places hence clients will not pay a significant transport fee [51]. Also, providing social support care such as transportation and childcare, having mobile clinics, and telemedicine can increase healthcare access by the clients [33]. Integration of methadone treatment in a general hospital setup can ensure early initiation of treatment and alleviate the stigma associated with treatment clinics situated separately [52, 53]. Some changes may involve change in clinic policies regarding take home doses, availing multiple MOUD to allow choice by clients, low threshold clinics and provision of MOUD in primary health care settings [32, 54, 55].

### Study strength and limitations

The strength of this study lies in being among the first studies to report experiences of clients receiving methadone treatment in Kenya. However, there are some limitations. Although an effort was made to recruit clients of different age groups, we could not recruit people older than 50. Therefore, we cannot gain an in-depth understanding of the experiences of older adults with opioid use and methadone treatment. In addition, findings from this study are based on one methadone treatment clinic in Nairobi and may not be generalizable to other areas. Because participants had limited time due to work responsibilities, they seemed in a hurry to complete the interviews, hence the short interviews. However, despite the short duration of interviews, participants were able to provide data that was insightful regarding their experiences.

## Conclusion

These findings provide insights into areas that can be addressed to improve the delivery of methadone treatment, given the benefits reported by those in treatment. Given the challenges clients face in seeking treatment for opioid use disorder, prevention initiatives should be emphasized. These include engaging at-risk communities in exploring ways to reduce early exposure to substances and early detection of substance use. For example, implementing prevention interventions in elementary schools, community education, and community-led substance use prevention interventions. There is also a need for future studies to explore the experiences of clients in other methadone treatment clinics and also get the view of clients after the introduction of the mobile clinic and prison delivery of methadone which were not present at the time the study was being conducted.

## Supplementary Information

**Additional file 1: Table S1** Overall format of semi-structured interviews guide used in the study.

## Data Availability

All data used and reported in this current study are available from the corresponding author upon reasonable request.

## Abbreviations

COVID-19: Coronavirus disease, 2019
DSM: Diagnostic and statistical manual of mental disorders
HIV/AIDS: Human immunodeficiency virus/acquired immunodeficiency syndrome
MOUD: Medications for opioid use disorder
OUD: Opioid use disorder
