# Supplementary material for: Exploring the experiences of clients receiving opioid use disorder treatment at a methadone clinic in Kenya: a qualitative study
Source: Addict Sci Clin Pract. 2022 Dec 12;17:71. doi: 10.1186/s13722-022-00352-z (PMC9742652; doi:10.1186/s13722-022-00352-z)
Supplement: Supplementary file 1 — Additional file 1: Table S1 Overall format of semi-structured interviews guide used in the study. [file 13722_2022_352_MOESM1_ESM.docx]

**Table S1: Overall format of semi-structured interviews guide used in the study**

| **Question** | **Guide for probing questions** |
| --- | --- |
| 1. Demographics | Age; sex; level of education: employment status; marital status |
| 1. Addiction history | - Which substances did you use prior to starting methadone treatment? - Any substance used currently? - At what age did you start any substance use? - At what age did you start heroin use? - Who introduced you to heroin? - What was the average amount of money spent on heroin before starting methadone - Have you sought treatment for them? |
| 1. Effect of methadone prior to starting methadone treatment | Do you think heroin use affected you in any way?  How, please explain |
| 1. Experiences with methadone treatment | - How and why did you get enrolled in the methadone treatment? - How long have you been on methadone treatment? - Do you know your current methadone dose? - How has it been for you to be on methadone? - How would you describe your experience at the clinic? - How has being on methadone affected your life? |
| 1. Adherence to methadone treatment | - How many doses have you missed in past one month? - What keeps you going? - Have you considered dropping out of treatment? - How would you like to be supported to remain in treatment? |
| 1. Support | - Who do you count on as a family and where are they located? - Where do you get your social support? - Do you face stigma of any kind? - Do you have a permanent address? |
| 1. Mental health | - Do you have unresolved mental health issues? - Are you on treatment for mental health disorder (depression, anxiety) |
| 1. What challenges do you face in methadone treatment? | Physical health; Mental health; Access to care; Stigma; System factors |
| 1. For those who had dropped out of treatment and were restarted in the program | - What made you relapse into opioid use when you were on methadone? - Is there anything you feel the clinic staff could have done to keep you from returning to substance use? - What made you come back to methadone treatment? |
| 1. What do you think can be done to improve methadone treatment? | How can the services at the clinic be improved? |
